# A Popular Treatise on the Kidney

**Published:** 1840-07

**Authors:** 


### ART. XII.

*A Popular Treatise on the Kidney; its hitherto Unknown Functions, and its Diseases in connexion with the Circulating Animal Oils, &c.* By GEORGE CORFE.—*London*, 1839. 8vo, pp. 304.

THIS work, from the pen of Mr. George Corfe, the resident medical officer to the Middlesex Hospital, is one of the most singular modern productions that it has been our lot to peruse. It may safely be pronounced a psychological curiosity, and would seem to have been thrown upon the present century by a sort of hap-hazard. It certainly can be classed only with the writings of the Theosophists and Cabbalists of the fifteenth and sixteenth centuries.

The work is written in a rather obscure style; and from its defective arrangement the views of the writer are understood only with much difficulty. It would be almost impossible as well as unprofitable to our readers to attempt anything like an analysis, but we may mention that it is divided into ten parts, under the titles of: 1. Divine Acceptance—ancient and modern writers on the Kidney. 2. Comparative Anatomy of the Kidney. 3. Anatomy; the Oil-tubes, &c. 4. Anatomy; the Blood. 5. The Circulating Animal Oils. 6. Animal Heat. 7. Salts, Alkalies, Acids, &c. Desultory Remarks. 8. Collateral Views of the Kidney. 9. Advice, &c. on Healthy Urine. 10. Diseases of the Kidney, &c. These parts are divided into chapters in a very arbitrary manner.

We shall give as brief and concise a view of the author's physiological doctrine of the kidneys as possible. The mass of fat in which the kidney is imbedded the author dignifies by the name of the "log of oil." Anatomists all know that the fat in question passes around the pelvis and vessels of the kidney, and that a portion of it also occupies the centre of the gland. No one that we are aware of has hitherto supposed that this fat performs any particular function in the secretion of urine, but such, according to Mr. Corfe, is the case. From the fat immediately surrounding the pelvis, seven divisions or branches pass off between the calyces, and these are denominated the "seven semicircular oil-tubes of the kidney." From the seven principal oil-tubes other smaller ones arise, which may perhaps vary from 80 to 100 in number.

"From the whole series of these large and small 'semicircular oil-tubes' come off an innumerable set of tubes, shaped with barbs and barbules, like a feather; I shall therefore term these 'THE FLOCCULENT FEATHERY OIL-TUBES' of the kidney. They may be described as coming off from the ends of the seven (semicircular oil-tubes), and as hanging about like so much down or shaggy substance when properly prepared for demonstration. They are dispersed throughout the whole medullary and vascular substance of the gland, and compose at least three fifths of the bulk of the organ. They may be traced to within one quarter of an inch of the surface of the kidney where they are lost in minute terminations." (p. 67.)

The "flocculent feathery oil-tubes" thus described, and of which engravings from the different divisions of the animal kingdom are given, the author believes to be the same with the "glandular ducts" of Bertin, the "white cortical ducts" of Ferrein, and the "serpentine ducts" of Müller.

Their essential character, according to Mr. Corfe, is that they are incapable of being injected either from the renal artery or vein or from the ureter.

Such are the new anatomical results at which the author, so far as we can collect, imagines he has arrived. We very much doubt, however, whether his views will receive the assent of anatomists; and though we have not reexamined the anatomy of the kidney, in relation to this subject, yet we will venture to question whether these "feathery oil-tubes" are anything else than an interstitial cellulo-vascular tissue analogous to the capsule of Glisson in the liver.

Let us now return to the physiology of the kidney as propounded by our author.

On this subject our views have hitherto been entirely wrong, according at least to Mr. Corfe, who shall speak for himself.

"The nature and office of the kidney is first to act in the highest temperature of animal heat, and to thrive in unctuous warmth......... Secondly, the office of the kidney is primarily, to receive oil within its bosom for the purposes of purifying that oil, and of lubricating the parts, and for the receiving of the DRAINS, or, as they are called, secretions from that oil, in order to discharge them in an excrementitious form from the animal economy. The office of the kidney is secondarily to receive the drains also, or as they are called, secretions from venous blood, and not from the rich life-giving arterial blood, as is universally supposed. And, thirdly, I maintain that the oil so purified then passes into the circulation by the trunks of the veins within the kidney and circulates through the system." (p. 29.)

The following is stated to be the mode in which the secretion of urine is accomplished. The "log of oil" before alluded to is stated to be

"A series of cells, all communicating one with another, and containing a large quantity of oil when warm, or suet, as we term it, when cold. The cells that are farthest from the kidney are the largest and the most dense of the whole 'log.' As the oil drops from cell to cell it passes through partitions thinner and thinner, or sieves finer and finer, until the network is so delicate around and within the gland, that it requires a magnifying power to demonstrate it. ........ If a small portion of oil is taken out of the adipose membrane most remote from the kidney, and smeared over the hand, it runs lumpy and hard over the skin. If a portion, however, be removed from the minute cells, just as it is entering into the bosom of the kidney, and similarly treated, it runs over the hand like tallow taken from under the flame of a candle. If the grosser lumps be taken, again, and held over a spirit lamp, it spurtles and burns with a crackling noise, as though it contained water and salt. But, on the contrary, a portion from the bosom of the kidney burns silently, rapidly, and is truly PURE OIL." (p. 64.)

The changes in the peri-renal fat, thus described, are stated by our author to depend on its excrementitious or watery and saline portions having been absorbed by the "flocculent feathery oil-tubes" in connexion with it. This excrementitious fluid becomes mixed in the urinary ducts with the secretion or drain from the *venous* blood of the kidney.

"The combined fluids then constitute urine, and not before." (p. 37.)

"The (renal) artery is merely a nutrient vessel and nothing else. It answers in all its characters to the hepatic or nutrient artery of the liver." (p. 77.)

The author observes that the fact of urine being discovered and pronounced to be a secretion from animal oil, will perhaps at first be received with something of a similar sensation to that occasioned by the discovery of the circulation by Harvey and that of vaccination by Jenner!

He lays two or three facts before the reader, that he may pause before any judgment is pronounced.

"First, that the tallow-makers out of 200 gallons of melted fat draw off upwards of nine gallons of water. Secondly, that, according to the researches of Chevreul and Braconnot, animal fat consists of a spermaceti-like substance, of oil, ammonia, potash, lime, and iron, which very substances are found under certain modifications in the urine. Thirdly, that the purified oil in the bosom of the kidney is found to have spent its watery and saline particles, and consequently burns silently." (p. 71.)

Various reasons are alleged at pages 88, 91, why the urine cannot be a secretion from arterial blood; but for these we must refer the reader to the work itself. The analogy of the liver is referred to in the following manner, as further corroborative of the views taken by the author:

"The physiology of the kidney exactly corresponds with my view of that of the liver. There are but few slight shadows of difference between them. Portal blood, from which bile is separated, is blood into the composition of which oil largely enters. This oil is derived from the caul, the mesentery, the intestines, and even, as I doubt not, from the blood itself. The portal blood is a fluid wholly for the secretion of bile, as Mr. Kiernan most ably demonstrates. The hepatic artery is exclusively a nutrient artery, and the hepatic vein receives both arterial and portal blood. The kidney, however [differs in having], its own distinct departments for the offices of secretion from oil and secretion from blood." (p. 79.)

Mr. Corfe "doubts not but that future researches will prove that the whole glandular system of secretion is effected through the media of oil and venous blood." (p. 89.)

Such are the doctrines which are propounded to us as a new physiology of the kidney. Without asserting that they are altogether destitute of plausibility, we must say that the author has altogether failed in establishing them.

We have gone over much tedious ground in order to present to our readers as condensed and correct a view of the author's doctrines as his ill-arranged book will allow of. Our principal object in doing so is to exhibit some of the devious paths into which a neglect of the inductive method of philosophizing will lead us.

It may perhaps be worth while to trace, so far as we are able, the source of this new physiology of the kidney. In the Mosaic ritual "the two kidneys and the fat that is upon them" (Exod. xxix., Levit. iii., &c.) formed an essential part of the various offerings which were ordained for the temple worship. Mr. Corfe in his first chapter accordingly quotes all the passages in which mention is made of these parts; and we have little doubt but that his book has originated in a desire to establish a pre-eminence of the "kidneys and their fat" in a physiological sense, which may correspond to their apparent spiritual and mystical pre-eminence. The following quotations we think support this assertion:

"The ALMIGHTY CREATOR of man has, in my mind, bestowed great honour on that inward part the kidney, in selecting it to be cut out of brutes and offered up on his own holy altar with the kindred fat and caul or midriff, as a typical sacrifice. Most important must that organ be in animal life which is so PICKED OUT BY THE DIVINE MAKER, who wound its every thread from his own finger." (p. 191.)

"By the kidneys it appears that the emblem of life, or the seat of vitality, is intended by the INFINITE MIND to be represented.

"When the LORD speaks of the quintessence of wheat, or its best and most valuable part, its life, its vital principle, then the LORD the SPIRIT expresses it by the kidneys of wheat, and the fat of the kidneys of wheat.—Deut. xxxii., Ps. cxi.-iii." (p. 187.)

Having in a previous place made the extraordinary statement that "vital substance is the kidney, and vital fluid the oil," (p. 114,) the author remarks:

"That very much indeed of the vital principle is locked up in the kidney of brute and rational animals, I believe, simply because I infer it from the eternal word of the GOD OF TRUTH and unerring wisdom. Men are, alas! so puffed up with earthly wisdom that they draw their inferences after the manner of carnal men. In proud contempt of all beside, they leave the mighty shadowings of GOD's almighty pencil in his tracery of the fabric of the human body, as beheld in His marvellous scriptures, by the microscopic eye of illuminated spiritual intellect, to the methodists, fanatics, and visionaries." (p. 188.)

Again,

"To my imagination the kidneys, or I shall say the kidney, sits a sovereign in animal life in the very centre of its kindred body. With one arm, as it were, she receives the oil, and with the other she rejects its refuse. The kidney sits with her back against the reins or loins, and hence she derives her strength; so in Christ all men have life, whether carnal or spiritual, &c. ....... She derives her moisture not so much from the multitude of oily channels within herself as from the gracious and copious insinuating anointings of the fine unctuous matter all around and about her. Have we not here some shadowings of the natural man? He has not by nature one atom of grace, yet that oil of rich mercy is copiously shed," &c. (p. 191.)

"It has been seen in some of the foregoing pages of this work that the peculiar secretion of the kidney is therein maintained to consist of parts drained off from oil and venous blood. It is most interesting to observe how the eternal word of truth appears to join these two important matters together. Oil and blood in type of blood and wine are continually placed side by side. Thus the fat of the kidney of wheat is close upon the blood of the grape.—Deut. xxxii." (p. 180.)

Passing over numerous other strange similitudes and analogies which Mr. Corfe's prolific "imagination" detects between things spiritual and natural, we shall conclude our notice of his work by observing that we never before met with so striking an illustration of Lord Bacon's remark, that "from the wild mixture of divine things with human, arises not only fantastical philosophies but heretical religions;" and again, "that men by supposing such a perfection in the scriptures, as that all philosophy should be derived from them, do not, as they imagine, honour but rather disgrace and pollute them."

